# Deep Inferior Epigastric Perforators Topography for “Island Transverse Rectus Abdominis Musculocutaneous Flap” in Breast Reconstruction

**DOI:** 10.1055/a-2093-8323

**Published:** 2023-08-02

**Authors:** Tae Hyun Kim, Seong Heum Jeong, Hee Chang Ahn

**Affiliations:** 1 Department of Plastic and Reconstructive Surgery, CHA Bundang Medical Center, CHA University Graduate School of Medicine, 59 Yatap-ro, Bundang-gu, Seongnam-si, Gyeonggi-do, Republic of Korea

**Keywords:** perforators, Island TRAM flap, breast reconstruction, topographic mapping

## Abstract

**Background** The Island transverse rectus abdominis musculocutaneous (TRAM) flap is well vascularized with very reliable blood flow, because all perforators of the zone I are included when it is harvested. The number of perforators, topographic mapping, and their relationship with reconstructed outcomes were investigated.

**Methods** Fifty patients with Island TRAM breast reconstruction from September 2021 to August 2022 were investigated. The zone I was divided into a total of eight sections. Under the loupe magnification, all perforators larger than 0.5 mm in zone I were counted with fine dissection, and photographs were taken in background of vessel loops. Complications like flap necrosis, seroma, and hematoma were also investigated.

**Result** There are 12 ideal perforators on average in zone I such as one perforator in section I, II, IV, V, VI, VIII, and three perforators in section III and VII. However, two perforators (M6 and L6) below arcuate line were sacrificed in the time of flap harvest to prevent hernia. Island TRAM included 10 perforators on average (5 perforators in each side) above arcuate line to be transferred to the recipient site. Only minor complications were identified.

**Conclusion** The Island TRAM flap includes 10 perforators to get the vigorous blood flow. The periumbilical to upper medial perforators become more dominant in the perfusion of the flap after deep inferior epigastric artery division. Well preserved perforators will guarantee the satisfactory breast reconstruction with the least complication.

## Introduction

Breast reconstruction methods after breast cancer resection are broadly divided into tissue expanders, permanent implants, and autologous tissue. Among them, the use of an abdominal tissue flap, introduced by Dr. Carl Hartrampf in 1980s, that is the transverse rectus abdominis myocutaneous flap (TRAM flap), 1 2 3 is still the most widely used method. Several standard options using free TRAM flap or deep inferior epigastric artery (DIEA) flap, including conventional TRAM flap, have been proposed, but pedicle TRAM surgery is considered first for the widest stable breast reconstruction. 4 5 6 7 8 9 10 11 12 The pedicled TRAM includes a method of importing flap from both the ipsilateral side and the contralateral side. The breast reconstruction that the flap is harvested from the ipsilateral side has several advantages, such as the inner inframammary fold can be preserved, the xiphoid shadow can be maintained, and the pedicle length can be long. In addition, due to these advantages, the flap movement is good so that the flap can be rotated according to the breast shape at times, and it has the advantage of excellent venous drainage.

Pedicle TRAM has benefits during and after surgery, which are relatively sufficient tissue can be obtained than other pedicled flaps, no postural changes during surgery, and a slim abdomen after surgery, resulting in increased patient satisfaction. In breast reconstructive surgery using pedicled TRAM flap, the importance of penetrating vessels from the superior epigastric artery (SEA) through the muscle to the flap has been emphasized all along, when DIEA is excised. Breast reconstruction surgery using Island TRAM flap has recently been studied and introduced as a novel flap with the advantages of free flap maintaining the shape of the inflammatory fold. There are three main reasons why the operation using the Island TRAM flap is evaluated as good: the rectus sheath can be directly closed, the shape of the inflammatory fold can be maintained without being affected as mentioned above, and the rotation of the flap is excellent by checking the position of the perforator. It is considered more important to preserve the blood vessels that originate from SEA to the flap as much as possible when the remaining muscles are finely dissectioned, except for the area where the upper abdominal wall artery enters the rectus abdominis muscle before bifurcation. 3

Even though there are numerous blood vessels that originate as transverse rectus muscle flaps, there has been no previously reported location of the perforator vessels that provide vigorous blood flow to their flaps. Therefore, in this study, the perforator that enables the maintenance of the healthy state of Island TRAM flap was identified during the surgery and topographic mapping of their location was created, which is considered very important and significant. The complication rate in this surgery was checked together to confirm and suggest the appropriate method of preserving the perforator as much as possible in breast reconstruction using TRAM flap.

## Methods

The surgery was conducted as a single-site study with approval from Institutional Review Board (IRB) medical institution. Breast reconstructions were performed using Island TRAM flap from September 2021 to August 2022, and patients who did not follow up for more than 3 months were excluded from the study. A total of 50 patients were included to confirm meaningful result. To introduce the surgical method using Island TRAM flap, the flap was designed to include approximately 2 cm above the navel, and the range of flap is zone I and zone III, which occupy most of the breast volume, but the tip of zone III was removed due to the possibility of complication such as fat necrosis. Additionally, some ranges of zone II that pass the navel have been included in flap's range.

The flap was harvested by fine dissection according to the design of the Island TRAM flap, and in this process all the perforators were identified as much as possible. Once all the ideal perforators larger than 0.5mm were found; occasionally, in the case of an ambiguous perforator, we tried to increase the accuracy of selection by using Doppler (DVM-3500). The vessel loop was used to record the position and number of ideal perforators on the medial side and the lateral side.

**Fig. 1:**
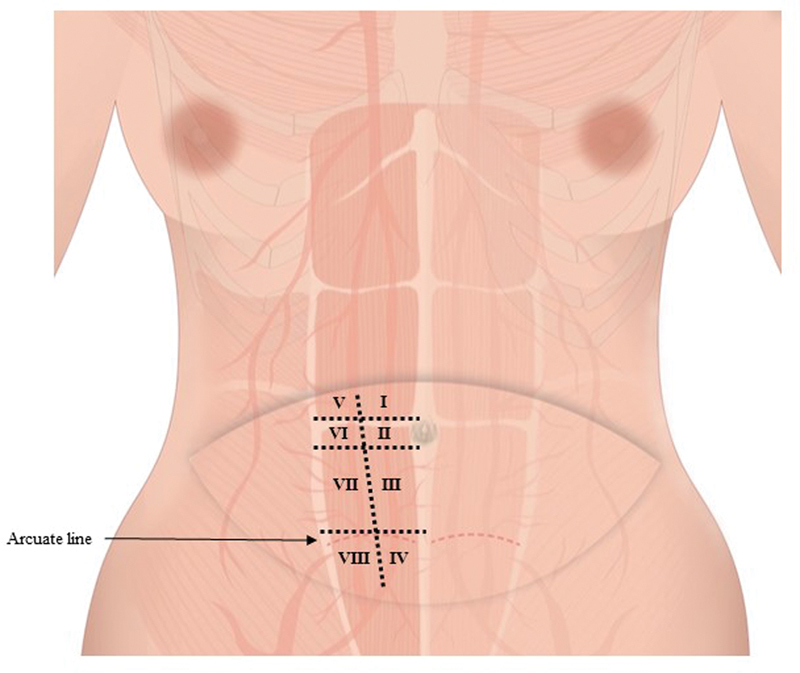
The zone I of the Island transverse rectus abdominis musculocutaneous flap was sectioned into eight sections. (4 areas such as upper, lateral, lower umbilical areas, and lower arcuate line were sectioned. Then these four areas were divided vertically in the center.)

**Fig. 2:**
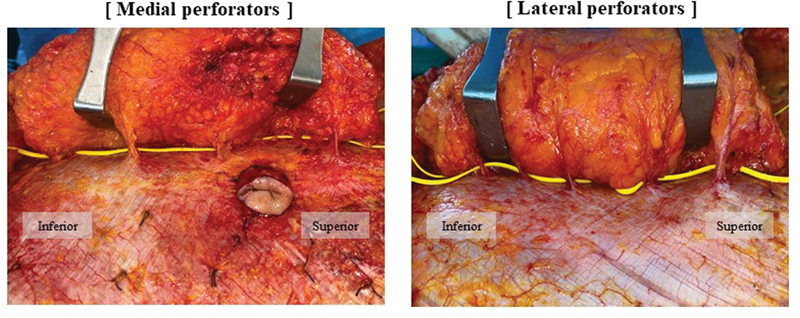
Under the loupe magnification, all perforators larger than 0.5 mm were finely dissected in the zone I. Then they were counted and topographically marked.

As a baseline, an imaginary line was drawn at the upper and lower border of navel, the arcuate lines, and symphysis pubis upper border to create a transverse baseline, and zone I was bisected into eight sections to the flap interface ( Fig. 1 .). The position of the perforator mentioned above was marked in each of these sections, and the ideal perforator was numbered from top to bottom in order on the medial side, namely M1, M2, and so on, and on the lateral side, they were similarly numbered in order from top to bottom as L1, L2, and so on. Only blood vessels with a thickness of 0.5 mm or more were considered as ideal perforators and exfoliation was performed ( Fig. 2 .)

**Fig. 3:**
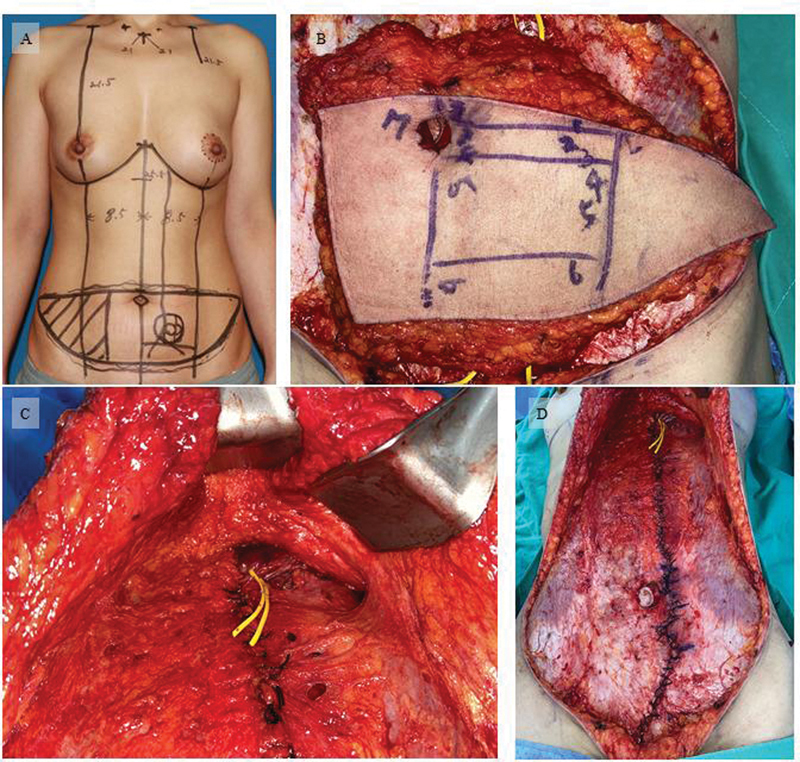
( **A)** The design before surgery and ( **B** ) the perforator and marks its location by classifying compartments according to criteria. ( **C** ) The superior epigastric artery was marked with a vessel loop to narrow the muscle pedicle. ( **D** )The direct closure of the fascia through Island transverse rectus abdominis musculocutaneous.

After finding as many as possible perforator of zone I that satisfies these criteria, the Z shape incision line was displayed using a marking pen for future repair of the fascia. An incision was made in the rectus fascia along the marked line, and rectus abdominis muscle was detached, while preserving the superior epigastric vessel with vessel loop. The muscle was excised near the arcuate line, de-epithelialized and fixed with silk threads. The flap was moved to the breast area through tunneling, and the remaining rectus muscle was pulled above the arcuate line and sutured to prevent hernia. The anterior rectus fascia was closed with 2-o silk ( Fig. 3 ).

A mapping was created by reviewing the photographic record of the recorded position of the perforator, and the most frequent section between each perforator was identified.

## Results

This study was conducted on a total of 50 patients. The average age was 52.02 years (30–64 years) and the average body mass index was confirmed to be 24.29. Only one patient was a smoker, seven patients had high blood pressure, and two patients had diabetes as an underlying medical condition. Regarding the history of abdominal surgery, 27 patients, more than half of the patients, underwent cesarean sections, and 12 patients underwent surgeries such as an appendectomy, a cholecystectomy, a hysterectomy, or an oophorectomy. A total of 36 patients underwent immediate breast reconstruction using Island TRAM flap, 13 patients underwent breast reconstruction as a delayed type, and 1 patient underwent surgery for cosmetic purpose. The types of mastectomy in patients who underwent mastectomy for noncosmetic breast cancer were also examined, with 16 patients receiving modified radical mastectomy, 17 patients undergoing skin sparing mastectomy, and 15 patients undergoing nipple sparing mastectomy. The average mastectomy specimen weight was 459 g. A total of 10 patients underwent neoadjuvant treatment, 8 patients underwent chemotherapy, and 2 patients underwent radiotherapy. Twenty patients underwent postoperative adjuvant treatment, 29 patients underwent radiation therapy, and 13 patients underwent hormonal therapy. Their average postoperative follow-up period was 282 days, approximately 8 to 9 months (122–445 days; Table 1 ).

**Table 1:** Patient demographics and perioperative surgical details of patients

Characteristic	Patients ( *n* = 50)
Age, y	52.02 (30–64)
Body mass index	24.29 (18.09–35)
Smoker	1 (2.0)
Hypertension	7 (14.0)
Diabetes	2 (4.0)
Abdominal operation history	
Cesarean section	27 (54.0)
Others	12 (24.0)
Types of mastectomy	
MRM	16 (32.0)
SSM	17 (34.0)
NSM	15 (30.0)
Mastectomy specimen weight, g	459.8 ± 152.4 (214–781.5)
Lymph node dissection	
SLND/ALND	33 (66.0) / 15 (30.0)
Types of breast reconstruction	
Immediate type	36 (72.0)
Delayed type	13 (26.0)
Augmentation mammoplasty	1 (2.0)
Neoadjuvant treatment	
Chemotherapy	8 (16.0)
Radiotherapy	2 (4.0)
Adjuvant treatment	
Chemotherapy	20 (40.0)
Hormonal therapy	13 (26.0)
Radiotherapy	29 (58.0)
Mean follow-up ± SD, days	282.6 ± 62.3 (122–445)

Abbreviations: ALND, axillary lymph node dissection; MRM, modified radical mastectomy; NSM, nipple-sparing mastectomy; SD, standard deviation; SLND, sentinel lymph node dissection; SSM, skin-sparing mastectomy.

When the ideal perforator identified during surgery was checked, based on the imaginary line bisected by zone I, an average of 5.86 perforators were identified on the medial side and 5.88 perforators on the lateral side ( Table 2 ).

**Table 2:** Number of the medial and lateral side perforators

Perforators	Patients ( *n* = 50)
Medial perforator (total)	5.86 ± 0.40
Lateral perforator (total)	5.88 ± 0.43

**Fig. 4:**
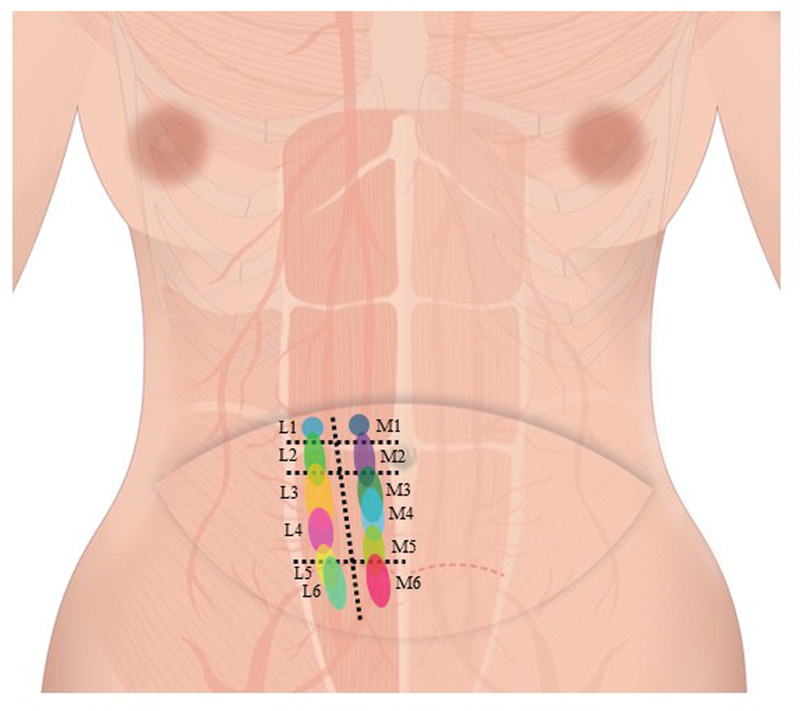
Topographic mapping for the distribution of perforator's location. Medial perforators (M1–6) and lateral perforators (L1–6).

**Fig. 5:**
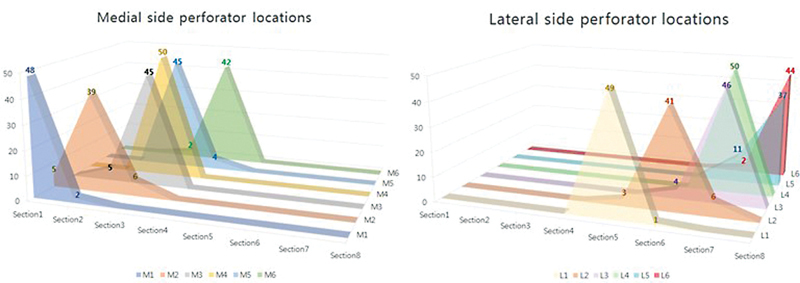
Distribution and number according to section of perforator on medial side and lateral side.

A detailed analysis of each section showed that M1, the medial side perforator, was identified in section I in 48 patients, accounting for 96%, and only two were identified in section II. In the case of M2, it was identified in section I in 5 patients, in section II in most of the patients (39), and in section III in 6 patients. When M3 was viewed, it was identified in section II in 5 patients, in section III in 45 patients, accounting for 90%. In the case of M4, it was found in section III in all patients. When M5 was identified, it was found in section III in 45 patients and in section IV in only 4 patients. Only one patient was found to have no M5 and only M4. In the case of M6, it was found in section III in two patients and in section IV in 42 patients (96%). When the perforator on the lateral side was checked, L1 was seen in section V in 49 patients except for one patient, and one patient showed it in section VI. For L2, it was found in section VI in 41 patients, in section V in 3 patients, and in section VII in 6 patients. Meanwhile, L3 was identified in section VII in 46 patients and in section VI in 4 patients. In the case of L4, it was all identified in section VII in all patients. L5 was present in section VII in 11 patients (23%) and in section VIII in the remaining 33 patients. In the case of L6, almost all of them, 44 patients had it in section VIII, and it was found in section VII only in 2 patients ( Table 3 , Fig. 4 , Fig. 5 ).

**Table 3:** Medial and lateral side perforators location analysis

Perforators	Sections							
	I	II	III	IV	V	VI	VII	VIII
Medial side perforators ( *n, %* )								
M1	48 (96)	2 (4)	0	0	0	0	0	0
M2	5 (10)	39 (78)	6 (12)	0	0	0	0	0
M3	0	5 (10)	45 (90)	0	0	0	0	0
M4	0	0	50 (100)	0	0	0	0	0
M5	0	0	45 (92)	4 (8)	0	0	0	0
M6	0	0	2 (4)	42 (96)	0	0	0	0
Lateral side perforators ( *n, %* )								
L1	0	0	0	0	49 (98)	1 (2)	0	0
L2	0	0	0	0	3 (6)	41 (82)	6 (12)	0
L3	0	0	0	0	0	4 (8)	46 (92)	0
L4	0	0	0	0	0	0	50 (100)	0
L5	0	0	0	0	0	0	11 (23)	37 (77)
L6	0	0	0	0	0	0	2 (4)	44 (96)

When the complication identified during follow-up period was confirmed, no patients required aggressive treatment for surgical site infection, except that one patient with hematoma who underwent additional ultrasound testing. Three patients, representing 6% of the study participants had seroma and wound dehiscence that required aspiration or additional revision procedures during follow-up period. In addition, there were no patients with hernia complication, and approximately 5 patients who partially showed small fat necrosis could be identified, but all of them were only focal and there was no patient with large fat necrosis. There were no patients with partial or total flap necrosis ( Table 4 ).

**Table 4:** Complication profiles of Island TRAM flap breast reconstruction

Complication	Nonirrigation ( *n* = 50) (%)
Surgical site infection	0 (0.0)
Hematoma	1 (2.0)
Seroma	3 (6.0)
Hernia	0 (0.0)
Wound dehiscence	3 (6.0)
Fat necrosis (small)	5 (10.0)
Fat necrosis (large)	0 (0.0)
Partial flap necrosis	0 (0.0)
Total flap necrosis	0 (0.0)

Abbreviation: TRAM, transverse rectus abdominis musculocutaneous.

## Discussion

Various methods of breast reconstruction have been proposed for a long time. In particular, in breast reconstruction using autologous tissue, the discovery and subsequent location of a perforator capable of supplying blood can lead to the continuous discovery of new flaps and the development of surgical methods, which in turn directly affects the improvement of the patient's quality of life and high satisfaction.

Although deep inferior epigastric perforator flap has recently been in the limelight because of difficulty in predicting optimal results due to muscle atrophy in TRAM flap, but TRAM flap breast reconstruction is still a safe, widely used autologous breast reconstruction method. The shape of the inflammatory fold can be affected by rectus muscle in conventional TRAM flap breast reconstruction. The Island TRAM flap surgery, which was introduced to improve this shortcoming and outcomes, has come to be considered a novel TRAM technique. 3 Therefore, research on nutrient vessel on TRAM flap to increase muscle fine dissection and flap mobility has been continued and the results have been presented.

According to Miller et al, in terms of choke vessels, approximately 60% of the vessels derived from the deep and upper abdominal walls are interconnected. These choke vessels are predominantly present between the third tendinous intersection of the rectus abdominis muscle and the tendinous intersection at the level of the navel. In other words, the connection between the lower abdominal wall blood vessels and the upper abdominal wall vessels begins just above the navel, so the importance of this area began to be emphasized. 11 According to Taylor et al, deep SEA, which is a terminal branch of the internal thoracic artery, and the branch of the external iliac artery, DIEA, are communicated at the umbilicus level of the rectus abdominis muscle. 11 However, we thought terminal branch of internal mammary artery was the main vessel to vigorous blood flow, and supply to get the blood to the Island TRAM flap, mainly. Meanwhile, according to the studies of Taylor and Daniel and Taylor et al, the DIEA pierces the transversalis fascia and enters the rectus sheath along the front of the arcuate line. 11 13

For subsequent branches, Milloy et al proposed the first major branching system in 1960. Tansatit et al and Ohjimi et al concluded that the main running of DIEA is a bifurcating system. 14 15 In addition, according to Moon and Taylor, it was suggested that this bifurcating system is numerically equivalent to approximately 57%. 16

Several studies have begun to suggest the number of musculocutaneous perforators originating from DIEA in hemiabdomen and the concept of “large” perforator, and in general, many studies have proposed the concept of “large perforator” in sizes greater than 0.5 mm or more than 1 mm and understand their numbers. They also checked where their general perforator concentration was located and found that they were mostly concentrated around the navel. 11 14 17 18 19 20 21 22 23 24 25 Vandevoort et al studied which form of these perforators was the most common and confirmed that the short intramuscular perforator type was the most common. 26

Kikuchi et al's study identified the most common overall perforator positions regardless of size, and set a grid of approximately 3 cm above the navel, 7 cm down, and 5 cm on each side to determine a total distribution of 329 perforators. 20 Their research is of great significance, which has confirmed the location of many perforators on a large scale. Although the advantage of using TRAM flap in breast reconstruction is that it can be properly positioned according to the breast shape, it must be preserved by providing vigorous blood flow to the flap among so many perforators, and the running distance of the blood vessel is at least 10 mm to increase mobility. When it was named “ideal perforator,” it only mentioned that there are about five of them in the hemiabdomen that must be preserved, but could not provide a mapping of their location.

As preoperative testing techniques have advanced, Leung et al were able to locate perforators using three-dimensional computed tomography, and Aubry et al have mentioned methods for identifying perforators before surgery, but they are not cost-effective for patients. So, there is a need for numbers and suggestions for the location of the ideal perforator in successful TRAM flap surgery. 27 28

In other words, knowledge of the position of the perforator is crucial for Island TRAM flaps because this technique involves using a section of the rectus abdominis muscle to reconstruct the breast. The Island TRAM flap technique preserves the blood supply to the tissue by using perforator vessels that travel through the muscle from the deep blood supply to the skin and fat of the abdomen. By identifying the location of the perforator vessels, the surgeon can ensure that the tissue used for reconstruction has a reliable blood supply, reducing the risk of complications such as flap loss or fat necrosis. However, the location of the ideal perforator of flap that enables the Island TRAM, which has been developed and considered a novel technique developed from it, including the conventional TRAM flap, has not been clearly confirmed and studied. In addition, it was not possible to summarize whether only about five ideal perforators were really present in the hemiabdomen. For the success of surgery, it is recommended to preserve as many perforators as possible, but since these studies were lacking, our study should be believed meaningful. In this study, the perforator location of 50 patients in the recently developed Island TRAM flap surgery was identified and created and presented the mapping accordingly. Analysis of the results showed that there were an average of 5.86 and 5.88 ideal perforators on the medial side and lateral side when patients were bisected from hemiabdomen to zone I. According to DIEA's bifurcating system, it was possible to see that there was an almost symmetrically similar number of perforators in each branch. It was found that section III and section VII had the largest number of perforators on average, where there were at least three ideal perforators in the space between the umbilicus lower border and the arcuate line. On the other hand, the thickest perforators among ideal perforators were mainly identified as M2 and M3, and these blood vessels were considered to be the main ideal perforator.

This study once again confirmed that perforator preservation in the periumbilical area is very important. Therefore, following this study, it was considered that it would be of great significance to additionally measure the blood flow of each perforator.

In breast reconstruction with Island TRAM flap, muscle resection is performed leaving enough lower rectus muscle to cover the arcuate line to prevent hernia, which is one of the common complications in conventional TRAM flap surgery. Then, musculocutaneous TRAM flap is transferred to the breast reconstruction area, in which M6 and L6 in sections IV and VIII are often not included in the Island TRAM flap. As a result, an average of 10 ideal perforators supply blood flow to Island TRAM flap. When these ideal perforators are well preserved, no major complications such as flap loss or large fat necrosis were noted, as can be seen from the complication results. Even minor complications such as hematoma, seroma, and wound dehiscence were rarely observed. And immediate concomitant nipple reconstruction successfully performed without the necrosis. This proves once again that strict preservation of an ideal perforator is important. The study of perforator location in the Island TRAM flap for breast reconstruction is an important research topic with significant clinical implications. This research can help surgeons identify the most reliable perforators and improve surgical techniques, which can lead to better outcomes for patients. Overall, this research provides valuable insights into the Island TRAM flap technique and highlights the potential for future development in this area, which can lead to improved surgical outcomes and better quality of life for breast cancer patients.

The Island TRAM flap differs from the standard TRAM flap procedure in that it preserves a small strip of muscle and blood vessels along with the skin and fat, creating a pedicle or “island” of tissue that remains attached to the original blood supply. This allows for greater flexibility in positioning the reconstructed breast and may help to reduce the risk of complications such as abdominal weakness or hernia. Through this study, the position of TRAM flap, as “ideal perforator” among the countless perforators in hemiabdomen in several studies, which is called “large perforator” and has a running distance of 10.0 mm or more flap that increases mobility, was identified. There were approximately 10 transferred ideal perforators in total, which provided vigorous blood flow and were confirmed by low complication rates. It could be confirmed why breast reconstruction with Island TRAM flap can be a novel TRAM technique as well as aesthetically satisfying. In particular, perforators in the periumbilical area are believed to be the main ideal perforator, and this study was able to suggest which position is important in breast reconstruction using Island TRAM flap.
